# Should Language Matter Less to Journals?

**DOI:** 10.1371/journal.pmed.0030246

**Published:** 2006-05-30

**Authors:** Erik von Elm

**Affiliations:** **1**University of BerneBerne, Switzerland University of Freiburg FreiburgGermany; **2**University of FreiburgFreiburgGermany

*PLoS Medicine* now encourages translations in languages other than English; this decision can only be welcomed.

German-speaking readers are pleased by the novelty of a PLoS title written in their language. The Editorial starts with the first line of the well-known German poem “Lorelei” by Heinrich Heine (1797–1856) [ 1]. This year commemorates the 150th anniversary of his death. Originating from Germany, Heine was an early European citizen and a mediator between cultures. After traveling through Europe for years, he settled down in Paris. There he wrote the “Lorelei” and other poems in German while his essays in French made German literature known to the francophone public.

In Heine's time, the question which language would prevail in science was yet undecided. In the 19th and the early 20th centuries pivotal works in different disciplines were published in languages other than English, as evidenced in the biographies of the Nobel Prize laureates ( http://nobelprize.org). It seems that insufficient knowledge of the one foreign language did not impede an academic career at that time.

Today, proficiency in English is a luxury for many users of scientific information, as pointed out in the Editorial. The same is true for the non-anglophone researchers, who invest time and money in English courses only to try to communicate with their anglophone peers on equal linguistic terms.

Can scientific journals do more to overcome the existing language barriers in the meantime? Sure, they can allow the translation of their papers in other languages. But would they also trouble to employ smart machines or humans in order to translate a manuscript written in one of Heine's languages into English before sending it out for peer review?

By the way, an English translation of the “Lorelei” is available at http://www.usd.edu/eric/deutsch/literatur/projekt/works/heine-lorelei.html.
